# Supplementary material for: MetaRibo-Seq measures translation in microbiomes
Source: Nat Commun. 2020 Jun 29;11:3268. doi: 10.1038/s41467-020-17081-z (PMC7324362; doi:10.1038/s41467-020-17081-z)
Supplement: Supplementary file 10 — Supplementary Data 7 [file 41467_2020_17081_MOESM10_ESM.zip › File2/Confidence_High_Taxonomy/93273_out.krona.html]

Javascript must be enabled to view this page.

members
magnitude
magnitudeUnassigned
count
unassigned
taxon
rank

93273\_out

3

superkingdom
2
3

976
3
phylum

class
117743
1

order
1
200644

49546
1
family

1
1016
genus

species
327575
1

SRS043729\_contig\_number\_12943

class
200643
2

171549
2
order

family
2
815

genus

SRS143876\_contig\_number\_contig-100\_31566.109001
2
1
816

626930
1

SRS043001\_contig\_number\_9057
species
